# Patients’ Characterization, Pattern of Medication Use, and Factors Associated with Polypharmacy: A Cross-Sectional Study Focused on Eight Units of the Portuguese National Network for Long-Term Integrated Care

**DOI:** 10.3390/healthcare12010057

**Published:** 2023-12-26

**Authors:** Catarina Candeias, Jorge Gama, Márcio Rodrigues, Amílcar Falcão, Gilberto Alves

**Affiliations:** 1 CICS-UBI—Health Sciences Research Centre, University of Beira Interior, Av. Infante D. Henrique, 6200-506 Covilhã, Portugal; 2 UMP—Union of Portuguese Mercies, Rua Entrecampos 9, 1000-151 Lisboa, Portugal; 3 CMA-UBI—Centre of Mathematics and Applications, Faculty of Sciences, University of Beira Interior, Rua Marquês D’Ávila e Bolama, 6201-001 Covilhã, Portugal; jgama@ubi.pt; 4 CPIRN-UDI-IPG—Center for Potential and Innovation of Natural Resources, Research Unit for Inland Development, Polytechnic Institute of Guarda, Av. Dr. Francisco de Sá Carneiro, 6300-559 Guarda, Portugal; 5 CIBIT—Coimbra Institute for Biomedical Imaging and Translational Research, University of Coimbra, Pólo das Ciências da Saúde, Azinhaga de Santa Comba, 3000-548 Coimbra, Portugal; acfalcao@ff.uc.pt; 6 Laboratory of Pharmacology, Faculty of Pharmacy, University of Coimbra, Pólo das Ciências da Saúde, Azinhaga de Santa Comba, 3000-548 Coimbra, Portugal; 7 ESALD-IPCB—Dr. Lopes Dias School of Health, Polytechnic Institute of Castelo Branco, Av. do Empresário, Campus da Talagueira, 6000-767 Castelo Branco, Portugal; 8 UFBI—Pharmacovigilance Unit of Beira Interior, University of Beira Interior, Av. Infante D. Henrique, 6200-506 Covilhã, Portugal

**Keywords:** older people, medications, polypharmacy, morbidity, Portugal

## Abstract

The Portuguese National Network for Long-term Integrated Care (RNCCI) comprises several Units for Integrated Continuous Care (UCCIs) that provide medical, nursing, and rehabilitation care. This study aimed to evaluate the demographic and medical characteristics of patients admitted to the RNCCI, their patterns of medication use, and factors associated with polypharmacy. An observational, retrospective, cross-sectional, multicenter study was performed. This study population consisted of 180 patients. Polypharmacy status was divided into two groups: non-polypharmacy (taking ≤ 4 drugs) and polypharmacy (taking ≥ 5 drugs). Bivariate analysis and multivariate logistic regression analysis were used to determine the influence of predictor factors such as demographic and medical characteristics on the polypharmacy status during the UCCI stays. This study population (mean age of 78.4 ± 12.3 years, range 23–102 years, 59% female) was prescribed a median of 8 medications. Approximately 89.4% of the patients were taking ≥ 5 drugs, demonstrating that polypharmacy is highly prevalent in Portuguese RNCCI residents of the eight UCCIs studied. A subsequent analysis with multivariate logistic regression found that polypharmacy status was significantly associated with the unit of internment (facility) when compared to facility E with H and with the Charlson Comorbidity Index (CCI). The high prevalence of polypharmacy and the associated factors show that it is urgent to improve pharmacotherapy regimens through periodic monitoring and review of patients’ therapeutic lists, an area in which pharmacists play a very important role.

## 1. Introduction

The progressive aging of the population is the result of multiple factors, two of the most important being the growth of average life expectancy and the increasingly low birth rate [1,2,3]. The increase in average life expectancy must necessarily be seen as a reflection of successful public health policies. However, it is true that these measures have also brought new challenges to society, which raises concerns about the sustainability of patient management and healthcare systems [3,4]. In fact, the aging of the population has led to changes in patients’ morbidity profiles, with a higher incidence of chronic diseases and a consequent greater demand on health and social care systems [3]. In this context, Portugal is no exception, presenting an average life expectancy of 81.3 years, which is slightly higher than the European Union average (80.9 years) [3,5]. Thus, considering the need for new policies to (re)configure health and social care, in 2006, the Portuguese authorities launched the National Network for Long-term Integrated Care (*Rede Nacional de Cuidados Continuados Integrados*, RNCCI). RNCCI is an integrated network of post-acute and long-term care units that resulted from a partnership between public, private, and third-sector entities [6,7] and that are currently available in Units for Integrated Continuous Care (*Unidades de Cuidados Continuados*, UCCIs). Its main goals are to help healthcare services improve patients’ transitions from hospital to home care, reduce the length of patients’ hospitalizations and avoid hospital readmissions, and also support people who require long-term care to deal with their mental, social, and physical limitations [6,8,9].

Epidemiologic studies suggest that multiple age-related diseases tend to be more prevalent in older people, leading to multimorbidity, polypharmacy, and a greater likelihood of developing adverse drug reactions (ADRs) and drug-related problems [10,11,12,13,14]. In particular, the ADRs are one of the foremost drug-related problems responsible for hospital admissions [15,16,17,18], healthcare costs [19,20], morbidity, and mortality [20,21,22,23,24,25,26], with many of them being associated with specific drug classes [21,23,27]. Still, it must be considered that around 50% of all ADRs could be prevented [18,22,28], including those associated with polypharmacy, an undesirable and expensive problem with potential negative clinical outcomes [29,30,31]. Although there is still a lack of consensus on the definition of polypharmacy, it is generally referred to as the concurrent use of multiple medications (i.e., ≥5 drugs) by the same individual [13,32]. Due to the progressive increase in the number of drugs concomitantly prescribed, several studies distinguish polypharmacy (defined as 5–9 drugs) from excessive polypharmacy (defined as ≥10 drugs) [33,34,35]. Furthermore, factors associated with polypharmacy and excessive polypharmacy have been explored [10,36,37]. Some studies on polypharmacy and patterns of medication use have been performed in different settings, such as nursing homes [38,39], hospital settings [36,40,41], and post-acute and long-term care settings [42,43,44,45,46]. However, despite the global tendency toward better healthcare for the population, no study to evaluate the patterns of medication use and the predictor factors such as the demographic and medical characteristics of polypharmacy have been conducted in Portugal, focusing on data from post-acute and long-term care residents from different UCCIs of the RNCCI.

Thus, this study aimed to evaluate and correlate the patients’ demographic and medical features with the pattern of medication use, prevalence of polypharmacy, and factors associated with patients from different UCCIs of the Portuguese RNCCI.

## 2. Materials and Methods

### 2.1. Study Design, Setting, and Population

An observational, retrospective, cross-sectional, and multicenter study was performed in UCCIs inserted in the Portuguese RNCCI. According to specific features, UCCIs of the RNCCI are currently divided into three different response typologies of hospitalization: (i) Convalescence Units (*Unidades de Convalescença*, UC) that provide medical, nursing, and rehabilitation care for stays up to 30 consecutive days; (ii) Medium-Term and Rehabilitation Units (*Unidades de Média Duração e Reabilitação*, UMDR) that offer less intensive nursing and rehabilitation care, with an expected length of stay between 30 and 90 consecutive days; and (iii) Long-Term and Maintenance Units (*Unidades de Longa Duração e Manutenção*, ULDM) that provide social support and maintenance healthcare for more than 90 consecutive days. This last response typology is specially intended for people with chronic diseases with different levels of dependency who are unable to be cared for at home, thus preventing and delaying the worsening of the dependency situation and favoring comfort and quality of life [6,47]. Different patients from UC, UMDR, and ULDM response typologies of hospitalization in the central region of Portugal were included in this study. To reduce bias associated with the type of hospitalization and the healthcare team, data were collected from one UC, four UMDR, and seven ULDM belonging to eight different UCCIs (A to H). The same number of patients (fifteen) from each UC, UMDR, or ULDM selected with a consecutive discharge date in the defined time period were selected. Data were collected considering the clinical processes at discharge.

The retrospective nature of this study did not affect healthcare provision to patients, and informed consent was not required. Patients’ data were anonymized through the attribution of an alphanumeric code, and access were restricted to the person who collected the data. The subsequent analysis were performed exclusively using the encoded data.

### 2.2. Data Sources

Data were mainly collected through the RNCCIs platform, which is an online tool that integrates multiple pieces of information about patients, such as medical, nursing, and social evaluations. This data system are regularly updated by the healthcare team, so it were used to collect most of the data on patients’ demographic records, medical history, diagnoses, and prescribed drugs; whenever available, additional internal records were also accessed to obtain information about the prescribed drugs.

### 2.3. Data Collection

For the eligible patients with the complete information records of the UCCIs selected, the following characteristics were collected by a pharmacist from the RNCCIs platform: demographic characteristics (age and gender), general information related to medical history (provenance/origin, length of stay, and type of feed), prescribed medications, and comorbidities. All pharmaceutical dosage forms, including oral, parenteral, topical, ophthalmological, and inhaled medications, taken on a regular basis (excluding only SOS medications), were considered. If a fixed-dose combination of drugs was used in the same medication, it was only counted as one. To describe the most frequently prescribed medications, drugs were grouped according to the Anatomical Therapeutic Chemical (ATC) classification system [48]: the first level of ATC classification (anatomical main group) and the second level of ATC classification (therapeutic subgroup); in both cases, whenever possible, the last update available (i.e., the prescription at discharge) was used. The polypharmacy status was classified as non-polypharmacy (≤4 drugs) and polypharmacy (≥5 drugs). Comorbidities were also investigated using the encoded diagnoses presented on the *GestCare CCI* platform. For this assessment, only diagnoses based on the International Classification of Diseases, Ninth Revision, and Clinical Modification (ICD-9-CM) were considered. Only the three ICD-9-CM codes existing in the patients’ profiles were collected, and only those that affected at least 5% of the total study population were reported. For the Charlson Comorbidity Index (CCI), all medical records were investigated [49].

### 2.4. Statistical Analysis

Continuous variables (age, length of stay, number of dispensed prescribed drugs, and CCI) were expressed as mean ± standard deviation, median, and interquartile range (P25; P75), and in the specific case of age, it also indicated the range. As for categorical variables, the number of observations (absolute frequency) and percentages (relative frequency) are explicitly shown. Logistic regression was performed to investigate the relationship between the main outcome (polypharmacy status) and the other variables [facilities (UCCIs, encoded from A to H); response typologies of hospitalization (UC, UMDR, and ULDM), demographic characteristics (age and gender), medical history (provenance/origin, length of stay, and type of feed), and CCI]. The existence of associations between the dependent variable and the evaluated independent variables was initially tested using bivariate logistic regression (an unadjusted model). The respective odds ratios (ORs) were also estimated and adjusted for possible confounding variables in a multivariate logistic regression (in which all the previously indicated variables were taken into account except the response typology of hospitalization). Logistic regression analysis with the logit link function was performed using the forward selection method based on the Wald test to find independent predictors associated with polypharmacy status. Also, ORs were adjusted for possible confounding variables. The Hosmer–Lemeshow test was performed to assess the goodness of fit, whereas the area under the receiver operating characteristic curve allowed the evaluation of the discriminatory power of the model and its sensitivity/specificity. The ORs were calculated considering a 95% confidence interval (CI). A *p*-value less than 0.05 (*p* < 0.05) was used as the significance level. Data analysis were performed using the statistical package IBM SPSS Statistics version 23 (IBM Corporation, Armonk, NY, USA) and GraphPad Prism software version 8.0 (San Diego, CA, USA).

## 3. Results

### 3.1. Characteristics of this Study Population

A total of 180 patients who received post-acute and long-term care at different response typologies from eight UCCIs of the RNCCI were included and extensively characterized. The characteristics of this study population are summarized in Table 1. Regarding demographic characteristics, this study population had a mean age of 78.4 ± 12.3 years in the range of 23–102 years, with the majority (59.4%) being female. Before being admitted to UCCIs, patients’ provenance/origin was mainly from hospital facilities (53.3%). For 53.3% of patients, the length of stay was longer than 90 days, and for 31.1%, it was between 31 and 90 days. Overall, 12.8% of patients had to be fed by enteral nutrition using a nasogastric tube or percutaneous endoscopic gastrostomy.

Regarding the number of dispensed prescribed drugs, patients had a median value of 8 (P25: 6; P75: 11) medications, with 38.9% having ten or more medications prescribed (Table 1).

To evaluate the most frequently prescribed drugs, 1594 prescriptions were initially considered. However, in some patients, there were repeated observations of drug prescriptions belonging to the same therapeutic subgroup, so it was considered that these should only be counted once, meaning that in the end, there were considered 1350 prescriptions. According to the ATC classification system, the main therapeutic subgroups prescribed were psycholeptics (67.2%), drugs for acid-related disorders (66.7%), antithrombotic agents (66.1%), psychoanaleptics (57.8%), diuretics (46.7%), agents acting on the renin-angiotensin system (40.0%), and lipid modifying agents (38.9%) (Table 1).

Concerning comorbidities, a total of 124 different ICD-9-CM codes were identified, with only those that affected at least 5% of this study population being selected. In addition, in 6 cases in which the ICD-9 code 436 (Acute, but ill-defined cerebrovascular disease) and the ICD-9-CM code 437 (Other and ill-defined cerebrovascular disease) were used, only the most recent diagnosis was considered. Thus, of the 234 comorbidities identified, only 228 were eligible for the final analysis. Overall, the results showed that approximately 29% of patients had essential hypertension, 26% had diabetes mellitus, and 13% were diagnosed with heart failure. The other prevalent identified conditions were acute (but ill-defined) cerebrovascular diseases, other cerebral degeneration, other and ill-defined cerebrovascular disease, fracture of the femoral neck, osteoarthrosis and related disorders, cardiac dysrhythmias, chronic kidney disease, and prostate hyperplasia (5%) (Table 1).

Regarding CCI, patients had a median value of 5 (P25: 4; P75: 7) (Table 1).

### 3.2. Factors Associated with Polypharmacy Status

**Figure 1:**
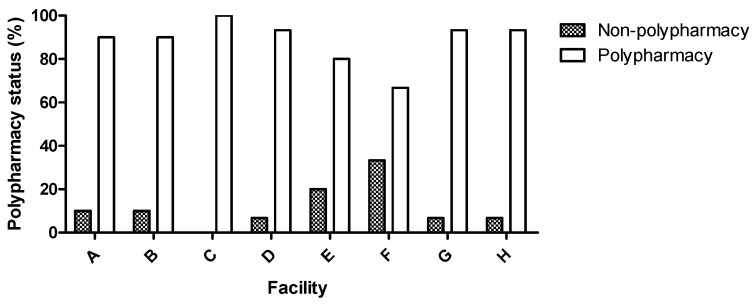
Polypharmacy status (non-polypharmacy and polypharmacy) according to the different facilities.

Table 2 summarizes the data related to polypharmacy status. Approximately 89.4% were subjected to polypharmacy (≥5 drugs), and only 10.6% of patients were prescribed less than 5 drugs. Among the different UCCI facilities (A to H), non-polypharmacy ranged from 0% to 33.3%, and polypharmacy varied between 66.7% and 100% (Figure 1 and Table 2). Considering the response typologies of hospitalization, 66.7%, 93.3%, and 90.5% of patients in the UC, UMDR, and ULDM, respectively, were subjected to polypharmacy regimens. In relation to age, the prevalence of polypharmacy was higher (92.4%) in the age group between 75 and 84 years old. Furthermore, 95.7% of patients fed by the enteral route were also subjected to polypharmacy. Regarding CCI, patients with higher scores were also more polymedicated.

Bivariate analysis identified as potential predictor factors of polypharmacy status: UCCIs [facility F when compared with H (OR = 0.143, 95%CI: 0.024–0.857; *p* = 0.033)]; response typologies of hospitalization [UMDR when compared to UC (OR = 7.000, 95%CI: 1.598–30.657; *p* = 0.010); ULDM when compared with UC (OR = 4.750; 95%CI: 1.353–16.675; *p* = 0.015)]; and CCI (OR = 1.424, 95%CI: 1.120–1.812; *p* = 0.004).

After multivariate logistic regression analysis (Table 3), a significant association was found between polypharmacy status and the unit of internment (facility) when facility E is compared with facility H (OR = 0.035, 95%CI: 0.003–0.417; *p* = 0.008). Polypharmacy status was also significantly associated with the CCI (OR = 1.914, 95% CI: 1.128–3.246; *p* = 0.016). However, no significant association was found with age, gender, or other factors assessed.

### 3.3. Distribution of Drug Users According to the Most Prescribed Drugs and Polypharmacy Status

Table 4 shows the distribution of drug users according to the most prescribed drugs and polypharmacy status, taking into consideration the anatomical main groups and therapeutic subgroups.

Regarding the anatomical main groups analysis, 751 drugs were considered to avoid the repetition of the counting of the same code in the same patient; of these, 98.0% (736 drugs) were included in the ten groups most frequently prescribed, and 93.5% (702 drugs) were reported by 10% of the total study population. In general, nervous system-active medications were the most chronically prescribed drugs (90.6%); drugs that act in the alimentary tract and metabolism and in the cardiovascular system had a similar prevalence (85.0% and 83.3%, respectively); and drugs belonging to the blood and blood-forming organs group also represented a significant part of the prescribed drugs (75.6%). When comparing the non-polypharmacy group with the polypharmacy group, the same trend can be seen between them, since the same anatomical main groups were those prescribed to the less medicated patients.

Regarding the therapeutic subgroup analysis, 1039 drugs belonging to the main 14 therapeutic subgroups were present in more than 20% of patients (Table 4). All of these therapeutic subgroups belong to the most commonly prescribed anatomical main groups (N, A, C, and B). Psycholeptics were prescribed to 42.1% of patients belonging to the non-polypharmacy group and to 70.2% of patients in the polypharmacy group. More than half of the patients with polypharmacy were prescribed at least a psycholeptic, a drug for acid-related disorders, an antithrombotic agent, or a psychoanaleptic drug.

## 4. Discussion

RNCCI in Portugal provides healthcare and social support to all patients in situations of dependency [7]. This support is given to patients in post-acute care that present a predictable end and, in an increasing way, to patients that may need lifelong, long-term care. Thus, this study analyzed patients from different UCCIs (A to H) that comprise the three response typologies (UC, UMDR, and ULMD), with a focus on their demographic and medical features, their pattern of medication use, the prevalence of polypharmacy, and other factors associated with these features.

This study population consisted of a representative sample of patients with a mean age of 78.4 ± 12.3 years, mostly female, with the majority having undergone long periods of hospitalization in the UCCIs. It was found that 8.3% of patients stayed in the UC (response typology for less than 30 days), 33.3% in the UMDR (response typology between 30 days and 90 days), and 58.4% stayed in the ULMD (response typology for more than 90 days). According to recent data, this obtained proportion are very close to the 10.3%, 28.5%, and 57.7% of the patients of national reality reported to be admitted to the UC, UMDR, and ULMD, respectively [50].

The described sample of patients was also analyzed in relation to the prescribed drugs. Around 90% of patients were found to be subject to polypharmacy (≥5 drugs), with a median value of 8 drugs per patient being prescribed. Regarding the classification of the prescribed drugs according to their anatomical main groups and therapeutic subgroups, it was found that the most frequently prescribed drugs belong to the nervous system (psycholeptics and psychoanaleptics), alimentary tract and metabolism (drugs for acid-related disorders), cardiovascular system (diuretics, agents acting on the renin-angiotensin system, and lipid-modifying agents), and also blood and blood-forming organs (antithrombotic agents). Our results are in agreement with the findings of other studies performed in nursing homes and in long-term care homes, in which the most prevalent therapeutic groups also involved the nervous system, alimentary tract, metabolism, and cardiovascular system [38,51]. Still, it is also important to note that other drugs such as nonsteroidal anti-inflammatory drugs [15,18,23,26,52] and antibiotics [52,53,54] have also been highly reported in the literature. However, those were not prevalent in our study, maybe because the data collected in our study refers to the period of discharge when patients are clinically stable. According to the literature, some of these therapeutic groups (e.g., cardiovascular agents [26,52,55,56], antidiabetics [26,27,52,55], analgesics [55], psycholeptics [26,52], diuretics [15,26,53], antithrombotics [21,23], and psychotropic drugs [21]) are described as predictors for ADRs.

The comorbidities (ICD-9-CM codes) most frequently found in our study (hypertension, heart failure, diabetes, osteoarthrosis, and allied disorders) have also been regularly reported in the literature: hypertension [57,58,59], heart failure [16,56,57], diabetes [16,56], renal and rheumatic diseases [16].

Our study calculated a polypharmacy prevalence of 66.7%, 93.3%, and 90.5% in the patients admitted to the UC, UMDR, and ULDM, respectively. These prevalence values were slightly higher than those found in the literature for older people in residence (67.4%), older outpatients (70%) [60], and nursing home residents (74%) [39], but similar to residents in long-term care homes [61], hospital patients (87.5%) [62], older patients discharged from hospital (85.9%) [63], and even older patients with urgent ADR-related hospital admissions (86%) [64]. Still, according to the literature, the prevalence of polypharmacy can differ widely between facilities [51,61], a statement that our results also recognized by observing that UCCIs themselves act as predictors for polypharmacy and high levels of polypharmacy. Therefore, it is suggested that periodic monitoring and drug prescription reviews could play an important role in reversing this trend.

By comparing facility E with facility H, we were able to identify a significant association between polypharmacy status and the unit of internment (facility). In addition to that, CCI was identified as a polypharmacy status predictor, probably because patients with more severe comorbid diseases may require more complex pharmacotherapeutic regimens to control their health status. In contrast, the other factors evaluated, such as age or gender, did not show statistically significant differences. Thus, healthcare professionals must pay special attention to patients with more comorbidities, which include pharmacists who are part of multidisciplinary teams where they could play an important role in medication reconciliation, preventing or reducing polypharmacy.

The present study also had some limitations, particularly the small size of the patient sample. Thus, to understand if the same medication pattern is maintained in the future, similar studies must be conducted in more healthcare centers. To identify the most frequent comorbidities, only diagnoses based on ICD-9-CM were considered, and only the three ICD-9-CM codes available were collected from patient profiles. It should also be considered that Portuguese UCCIs have a type of prescription policy that prefers the use of single-drug formulations instead of fixed-dose combinations of drugs and aims for easy dose adjustment whenever necessary. This practice can overestimate the results and contribute to a higher prevalence of polypharmacy.

## 5. Conclusions

Our investigation expands the knowledge on demographical and medical characteristics, patterns of medication use, and polypharmacy, as well as its predictor factors, in the Portuguese RNCCI, where data in this field are scarce. Our findings suggest that the studied population (patients with a mean age of 78.4 ± 12.3 years, a range of 23–102 years, and 59% female) was prescribed a median of 8 medications. Around 90% of patients were found to be subject to polypharmacy (≥5 drugs), and the most frequent anatomical main groups were the nervous system, alimentary tract and metabolism, cardiovascular system, and also blood and blood-forming organs. In addition, this study demonstrates that polypharmacy is highly prevalent in Portuguese RNCCI residents and is significantly associated with the facility (E vs. H) and with CCI. The higher prevalence of polypharmacy and its associated factors may indicate that, to achieve an optimal risk-benefit relationship in each patient’s therapeutic list, it is urgent to improve patients’ pharmacotherapy regimens through periodic monitoring and review of their therapeutic lists, an area in which pharmacists are in a unique position within the multidisciplinary healthcare teams belonging to the RNCCI. Hence, further research on drug use in which interventions by health professionals are performed as well as the impact of these interventions on post-acute and long-term care patients is needed to improve drug therapy.

## Figures and Tables

**Table 1:** Characteristics of this study population (*N* = 180) that received post-acute care and long-term care in Units for Integrated Continuous Care (UCCI) inserted in the Portuguese National Network for Long-term Integrated Care (RNCCI).

	Total (*N* = 180)	UC (*n* = 15)	UMDR (*n* = 60)	ULMD (*n* = 105)
**Demographic characteristics**				
Age (years)				
Mean ± SD (range)	78.4 ± 12.3 (23–102)	70.5 ± 13.6 (44–82)	77.3 ± 10.5 (34–94)	80.1 ± 12.6 (23–102)
Median (P25; P75)	81 (74.25; 86.00)	75 (62; 81)	79 (74; 83)	83 (75; 87)
<65, *n* (%)	19 (10.6)	4 (21.1)	7 (36.8)	8 (42.1)
65–74, *n* (%)	26 (14.4)	2 (7.7)	9 (34.6)	15 (57.7)
75–84, *n* (%)	79 (43.9)	9 (11.4)	32 (40.5)	38 (48.1)
≥85, *n* (%)	56 (31.1)	0 (0.0)	12 (21.4)	44 (78.6)
Gender, n (%)				
Male	73 (40.6)	6 (8.7)	24 (32.9)	43 (58.9)
Female	107 (59.4)	9 (8.1)	36 (33.7)	62 (57.9)
**Medical history**				
Provenance/origin, *n* (%)				
Residence or other	84 (46.7)	1 (1.2)	23 (27.4)	60 (71.4)
Hospital	96 (53.3)	14 (14.6)	37 (38.5)	45 (46.9)
Length of stay				
Mean ± SD	145.3 ± 189.5	30.5 ± 11.0	94.7 ± 49.3	190.7 ± 234.5
Median (P25; P75)	93 (59.25;150.00)	30 (30; 38)	92.5 (68; 115)	117 (86; 184)
≤30, *n* (%)	28 (15.6)	11 (39.3)	7 (25.0)	10 (35.7)
31–90, *n* (%)	56 (31.1)	4 (7.1)	22 (39.3)	30 (53.6)
>90, *n* (%)	96 (53.3)	0 (0.0)	31 (32.3)	65 (67.7)
Type of feed				
Enteral nutrition, *n* (%)				
No	157 (87.2)	15 (9.6)	54 (34.4)	88 (56.1)
Yes	23 (12.8)	0 (0.0)	6 (26.1)	17 (73.9)
Nasogastric tube, *n* (%)	20 (11.1)	0 (0.0)	6 (30.0)	14 (70.0)
Percutaneous endoscopic gastrostomy, *n* (%)	3 (1.7)	0 (0.0)	0 (0.0)	3 (1.7)
**Number of dispensed prescribed drugs**				
Mean ± SD	8.8 ± 3.6	5.9 ± 3.0	9.3 ± 3.0	8.9 ± 3.8
Median (P25; P75)	8 (6; 11)	6 (4; 8)	8.5 (7; 11)	9 (6; 11)
≤4, *n* (%)	19 (10.6)	5 (26.3)	4 (21.1)	10 (52.6)
5–9, *n* (%)	91 (50.6)	8 (8.8)	29 (31.9)	54 (59.3)
≥10, *n* (%)	70 (38.9)	2 (2.9)	27 (38.6)	41 (58.6)
**Most frequent prescribed therapeutic subgroups ^†^, *n* (%)**				
Psycholeptics (N05)	121 (67.2)	9 (7.4)	42 (34.7)	70 (57.9)
Drugs for acid-related disorders (A02)	120 (66.7)	11 (9.2)	42 (35.0)	67 (55.8)
Antithrombotic agents (B01)	119 (66.1)	10 (8.4)	42 (35.3)	67 (56.3)
Psychoanaleptics (N06)	104 (57.8)	9 (8.7)	33 (31.7)	62 (59.6)
Diuretics (C03)	84 (46.7)	4 (4.8)	32 (38.1)	48 (57.1)
Agents acting on the renin-angiotensin system (C09)	72 (40.0)	5 (6.9)	30 (41.7)	37 (51.4)
Lipid-modifying agents (C10)	70 (38.9)	5 (7.1)	25 (35.7)	40 (57.1)
Analgesics (N02)	59 (32.8)	4 (6.8)	19 (32.2)	36 (61.0)
Drugs for constipation (A06)	58 (32.2)	0 (0.0)	22 (37.9)	36 (62.1)
Beta-blocking agents (C07)	53 (29.4)	2 (3.8)	24 (45.3)	27 (50.9)
Drugs used in diabetes (A10)	51 (28.3)	1 (2.0)	24 (47.1)	26 (51.0)
Antiepileptics (N03)	47 (26.1)	2 (4.3)	14 (29.8)	31 (66.0)
Antianemic preparations (B03)	41 (22.8)	0 (0.0)	15 (36.6)	26 (63.4)
Cardiac therapy (C01)	39 (21.7)	0 (0.0)	14 (35.9)	25 (64.1)
**Most common/significant comorbidities (ICD-9-CM codes ^‡^), *n* (%)**				
Essential hypertension (401)	52 (28.9)	4 (7.7)	20 (38.5)	28 (53.8)
Diabetes mellitus (250)	47 (26.1)	0 (0.0)	23 (48.9)	24 (51.1)
Heart failure (428)	23 (12.8)	0 (0.0)	11 (47.8)	12 (52.2)
Acute, but ill-defined, cerebrovascular disease (436)	20 (11.1)	0 (0.0)	11 (55.0)	9 (45.0)
Other cerebral degenerations (331)	16 (8.9)	0 (0.0)	4 (25.0)	12 (75.0)
Other and ill-defined cerebrovascular diseases (437)	14 (7.8)	0 (0.0)	1 (7.1)	13 (92.9)
Fracture of the neck of the femur (820)	14 (7.8)	4 (28.6)	5 (35.7)	5 (35.7)
Osteoarthrosis and allied disorders (715)	12 (6.7)	6 (50.0)	0 (0.0)	6 (50.0)
Cardiac dysrhythmias (427)	11 (6.1)	0 (0.0)	6 (54.5)	5 (45.5)
Chronic kidney disease (585)	10 (5.6)	0 (0.0)	4 (40.0)	6 (60.0)
Hyperplasia of the prostate (600)	9 (5.0)	0 (0.0)	2 (25.0)	6 (75.0)
**CCI**				
Mean ± SD	5.5 ± 2.1	3.3 ± 1.9	5.5 ± 1.9	5.8 ± 2.0
Median (P25; P75)	5 (4; 7)	4 (2; 5)	5.5 (4; 7)	6 (5; 7)

CCI, Charlson Comorbidity Index; ICD-9-CM, International Classification of Diseases, Ninth Revision, Clinical Modification; SD, Standard deviation; UC, Convalescence Units; ULDM, Long-Term and Maintenance Units; UMDR, Medium-Term and Rehabilitation Units; **^†^** the therapeutic subgroups present in more than 20% of patients; **^‡^** ICD-9-CM codes affected at least 5% of the total study population.

**Table 2:** Factors associated with polypharmacy status (non-polypharmacy and polypharmacy) were subjected to a bivariate logistic regression (unadjusted model).

	Total *N* (%)	Non-Polypharmacy *n* (%)	Polypharmacy *n* (%)	OR ^†^ (95% CI)	*p* *
	180	19 (10.6)	161 (89.4)		
**Facilities**					
UCCI					0.290
A	30 (16.7)	3 (10.0)	27 (90.0)	0.643 (0.100; 4.153)	0.643
B	30 (16.7)	3 (10.0)	27 (90.0)	0.643 (0.100; 4.153)	0.643
C	15 (8.3)	0 (0.0)	15 (100.0)	-	-
D	30 (16.7)	2 (6.7)	28 (93.3)	1.000 (0.131; 7.605)	1.000
E	15 (8.3)	3 (20.0)	12 (80.0)	0.286 (0.042; 1.935)	0.199
F	15 (8.3)	5 (33.3)	10 (66.7)	**0.143 (0.024; 0.857)**	**0.033**
G	15 (8.3)	1 (6.7)	14 (93.3)	1.000 (0.083; 11.998)	1.000
H	30 (16.7)	2 (6.7)	28 (93.3)	1	
Response typology of hospitalization					**0.020**
UC	15 (8.3)	5 (33.3)	10 (66.7)	1	
UMDR	60 (33.3)	4 (6.7)	56 (93.3)	**7.000 (1.598; 30.657)**	**0.010**
ULDM	105 (58.4)	10 (9.5)	95 (90.5)	**4.750 (1.353; 16.675)**	**0.015**
**Demographic characteristics**					
Age (years)					
Mean ± SD (range)	78.4 ± 12.3 (23–102)	75.7 ± 18.0 (44–102)	78.7 ± 11.4 (23–99)	1.018 (0.983; 1.053)	0.322
Median (P25; P75)	81 (74; 86)	79 (58; 91)	81 (75; 86)		
<75, *n* (%)	45 (25.0)	7 (15.6)	38 (84.4)	1	
75–84, *n* (%)	79 (43.9)	6 (7.6)	73 (92.4)	2.241 (0.703; 7.141)	0.172
≥85, *n* (%)	56 (31.1)	6 (10.7)	50 (89.3)	1.535 (0.477; 4.962)	0.472
Gender					
Male	73 (40.6)	11 (15.1)	62 (84.9)	1	
Female	107 (59.4)	8 (7.5)	99 (92.5)	2.196 (0.837; 5.760)	0.110
**Medical history**					
Provenance/origin					
Residence or other (%)	84 (46.7)	11 (13.1)	73 (86.9)	1	
Hospital (%)	96 (53.3)	8 (8.3)	88 (91.7)	1.658 (0.633; 4.338)	0.303
Length of stay:					
Mean ± SD	145.3 ± 189.5	96.0 ± 76.4	151.1 ± 198.0	1.003 (0.998; 1.009)	0.248
Median (P25; P75)	93 (59; 150)	90 (30; 162)	94 (64.5; 150)		
≤ 30, *n* (%)	28 (15.6)	6 (21.4)	22 (78.6)	1	
31–90, *n* (%)	56 (31.1)	4 (7.1)	52 (92.9)	3.545 (0.910; 13.811)	0.068
> 90, *n* (%)	96 (53.3)	9 (9.4)	87 (90.6)	2.636 (0.848; 8.194)	0.094
Type of feed					
Enteral nutrition (%)					
Yes	23 (12.8)	1 (4.3)	22 (95.7)	2.849 (0.362; 22.426)	0.320
No	157 (87.2)	18 (11.5)	139 (88.5)	1	
**CCI**					
Mean ± SD	5.5 ± 2.1	4.2 ± 2.5	5.6 ± 1.9	**1.424 (1.120; 1.812)**	**0.004**
Median (P25; P75)	5 (4; 7)	5 (1; 6)	6 (4; 7)		

CCI, Charlson Comorbidity Index; SD, Standard deviation; UC, Convalescence Units; UCCI, Units for Integrated Continuous Care; ULDM, Long-Term and Maintenance Units; UMDR, Medium-Term and Rehabilitation Units; ^†^ Not adjusted odd ratio; * Wald test; All significant variables are in bold.

**Table 3:** Factors associated with polypharmacy status (non-polypharmacy and polypharmacy) were subjected to multivariate logistic regression (adjusted model).

	aOR ^†^ (95% CI)	*p* *
**Facilities**		
UCCI		0.109
A	0.686 (0.096; 4.895)	0.707
B	0.818 (0.110; 6.060)	0.844
C	-	-
D	1.073 (0.121; 9.553)	0.949
E	**0.035 (0.003; 0.417)**	**0.008**
F	0.133 (0.012; 1.409)	0.094
G	1.081 (0.078; 15.078)	0.954
H	1	
**Demographic characteristics**		
Age (years)	0.931 (0.867; 1.000)	0.051
Gender		
Male	1	
Female	2.253 (0.681; 7.458)	0.183
**Medical History**		
Provenance/origin		
Residence or other (%)	1	
Hospital (%)	4.369 (0.969; 19.698)	0.055
Length of stay	1.003 (0.997; 1.009)	0.310
Type of feed		
Enteral nutrition (%)		
Yes	1.739 (0.136; 22.269)	0.671
No	1	
**CCI**	**1.914 (1.128; 3.246)**	**0.016**

CCI, Charlson Comorbidity Index; ^†^ aORs, adjusted odd ratio with all the variables of Table 2, except with the response typology of hospitalization; *p* < 0.05 is significant; all significant variables are in bold. Omnibus test: *p* = 0.004; Hosmer and Lemeshow test: *p* = 0.683; Cox and Snell r^2^ = 0.16, Nagelkerke r^2^ = 0.32; AUC = 0.842 (95% CI (0.759; 0.925), *p* < 0.001); Sensitivity = 59.6%; Specificity = 94.7% (cutoff probability = 0.945); * Wald test.

**Table 4:** Distribution of drug users according to the most prescribed drugs and polypharmacy status.

	ATC Code	Total *N* (%)	Non-Polypharmacy *n* (%)	Polypharmacy *n* (%)
		180 (100%)	19 (10.6)	161 (89.4)
**Anatomical main groups ^†^**				
Nervous system	N	163 (90.6)	13 (8.0)	150 (92.0)
Alimentary tract and metabolism	A	153 (85.0)	10 (6.5)	143 (93.5)
Cardiovascular system	C	150 (83.3)	10 (6.7)	140 (93.3)
Blood and blood forming organs	B	136 (75.6)	9 (6.6)	127 (93.4)
Respiratory system	R	40 (22.2)	2 (5.0)	38 (95.0)
Musculo-skeletal system	M	34 (18.9)	0 (0.0)	34 (100)
Genito urinary system and sex hormones	G	26 (14.4)	0 (0.0)	26 (100)
Systemic hormonal preparations	H	15 (8.3)	0 (0.0)	15 (100)
Anti-infectives for systemic use	J	12 (6.7)	0 (0.0)	12 (100)
Dermatologicals	D	7 (3.9)	0 (0.0)	7 (100)
**Therapeutic subgroups ^‡^**				
Psycholeptics	N05	121 (67.2)	8 (6.6)	113 (93.4)
Drugs for acid related disorders	A02	120 (66.7)	8 (6.7)	112 (93.3)
Antithrombotic agents	B01	119 (66.1)	8 (6.7)	111 (93.3)
Psychoanaleptics	N06	104 (57.8)	5 (4.8)	99 (95.2)
Diuretics	C03	84 (46.7)	4 (4.8)	80 (95.2)
Agents acting on the renin-angiotensin system	C09	72 (40.0)	2 (2.8)	70 (97.2)
Lipid modifying agents	C10	70 (38.9)	4 (5.7)	66 (94.3)
Analgesics	N02	59 (32.8)	2 (3.4)	57 (96.6)
Drugs for constipation	A06	58 (32.2)	2 (3.4)	56 (96.6)
Beta blocking agents	C07	53 (29.4)	0 (0.0)	53 (100)
Drugs used in diabetes	A10	51 (28.3)	0 (0.0)	51 (100)
Antiepileptics	N03	47 (26.1)	2 (4.3)	45 (95.7)
Antianemic preparations	B03	41 (22.8)	1 (2.4)	40 (97.6)
Cardiac therapy	C01	39 (21.7)	1 (2.6)	38 (97.4)

**^†^** the ten most frequently prescribed anatomical main groups; **^‡^** the therapeutic subgroups present in more than 20% of patients.

## Data Availability

The datasets used and/or analyzed during the current study are available from the corresponding author upon reasonable request.
